# Optical Coherence Tomography in Myocardial Infarction Management: Enhancing Precision in Percutaneous Coronary Intervention

**DOI:** 10.3390/jcm13195791

**Published:** 2024-09-28

**Authors:** Angela Buonpane, Giancarlo Trimarchi, Marco Ciardetti, Michele Alessandro Coceani, Giulia Alagna, Giovanni Benedetti, Sergio Berti, Giuseppe Andò, Francesco Burzotta, Alberto Ranieri De Caterina

**Affiliations:** 1 Department of Cardiovascular Sciences, Fondazione Policlinico Universitario A. Gemelli IRCCS, Università Cattolica Sacro Cuore, Rome, Largo Agostino Gemelli, 1, 00168 Roma, Italy; buonpaneangela@gmail.com (A.B.); francesco.burzotta@unicatt.it (F.B.); 2 Department of Clinical and Experimental Medicine, University of Messina, 98100 Messina, Italy; giancarlo.trimarchi18@gmail.com (G.T.); giulia.alagna@icloud.com (G.A.); 3 Interdisciplinary Center for Health Sciences, Scuola Superiore Sant’Anna, 56127 Pisa, Italy; 4 Cardiology and Pneumology Division, Fondazione Toscana G. Monasterio, 56124 Pisa, Italy; mciard@ftgm.it (M.C.); michecoc@ftgm.it (M.A.C.); 5 Fondazione Toscana G. Monasterio, Ospedale del Cuore G., Pasquinucci, 54100 Massa, Italy; giovanni.benedetti@ftgm.it (G.B.); ifcberti@ftgm.it (S.B.); adecaterina@ftgm.it (A.R.D.C.)

**Keywords:** acute myocardial infarction, optical coherence tomography, mechanisms of acute coronary syndrome, precision medicine

## Abstract

In acute myocardial infarction (AMI), the urgency of coronary revascularization through percutaneous coronary intervention (PCI) is paramount, offering notable advantages over pharmacologic treatment. However, the persistent risk of adverse events, including recurrent AMI and heart failure post-revascularization, underscores the necessity for enhanced strategies in managing coronary artery disease. Traditional angiography, while widely employed, presents significant limitations by providing only two-dimensional representations of complex three-dimensional vascular structures, hampering the accurate assessment of plaque characteristics and stenosis severity. Intravascular imaging, specifically optical coherence tomography (OCT), significantly addresses these limitations with superior spatial resolution compared to intravascular ultrasound (IVUS). Within the context of AMI, OCT serves dual purposes: as a diagnostic tool to accurately identify culprit lesions in ambiguous cases and as a guide for optimizing PCI procedures. Its capacity to differentiate between various mechanisms of acute coronary syndrome, such as plaque rupture and spontaneous coronary dissection, enhances its diagnostic potential. Furthermore, OCT facilitates precise lesion preparation, optimal stent sizing, and confirms stent deployment efficacy. Recent meta-analyses indicate that OCT-guided PCI markedly improves safety and efficacy in revascularization, subsequently decreasing the risks of mortality and complications. This review emphasizes the critical role of OCT in refining patient-specific therapeutic approaches, aligning with the principles of precision medicine to enhance clinical outcomes for individuals experiencing AMI.

## 1. Introduction

Coronary artery disease (CAD) remains a primary global health concern, with acute myocardial infarction (AMI) standing out as one of the most frequent manifestations of ischemic heart disease [1]. Despite the widespread implementation of invasive treatment strategies, certain patient groups continue to exhibit a heightened risk for subsequent cardiovascular events [2]. Consequently, significant research efforts over the past few decades have focused on identifying novel risk factors and enhancing diagnostic, pharmacological, and invasive interventions for AMI [3,4,5,6,7,8,9,10,11]. In cases of AMI, coronary angiography is typically the initial diagnostic tool, facilitating revascularization and stenting of the affected coronary artery [12]. Although coronary angiography has long been considered the gold standard for evaluating the presence, location, and severity of CAD, it is limited in its ability to provide insights into plaque composition and biological activity [13]. As a two-dimensional (2-D) representation of a three-dimensional (3-D) vascular structure, angiography falls short in accurately characterizing plaque, vessel walls, and lumen metrics [14]. This inadequacy becomes particularly evident in complex lesions (bifurcations or left main disease) where procedural success and long-term outcomes may not meet expectations [15,16]. Even following successful revascularization, patients remain susceptible to recurrent AMI, heart failure, and stent thrombosis, underscoring the necessity for improvements in the strategies and techniques used in coronary revascularization [17].

In this context, intravascular imaging has emerged as a pivotal tool, enhancing clinical outcomes through the precise evaluation of coronary vessels, even in patients suffering from AMI [18]. This advanced imaging approach addresses the inherent limitations associated with standard coronary angiography. Prominent modalities include intravascular ultrasound (IVUS), near-infrared spectroscopy IVUS (NIRS-IVUS), and optical coherence tomography (OCT). Each of these methods provides distinct benefits for visualizing and evaluating the lumen, the structure of the vessel, and atherosclerotic plaque phenotypes [19].

IVUS is instrumental in providing comprehensive pre-intervention data on lesion characteristics, such as plaque morphology and vessel dimensions, boasting a resolution between 100 and 200 micrometers—superior to that of angiography. Multiple randomized studies have demonstrated that the use of IVUS as guidance for stenting results in better clinical outcomes compared to angiography-guided approaches [20]. 

On the other hand, OCT delivers an exceptional resolution of 10 micrometers, allowing for the meticulous assessment of the coronary plaque phenotype and providing critical post-intervention information regarding stent expansion and apposition and stent edge dissections [21]. While both IVUS and OCT utilize intracoronary imaging catheters, they present distinct characteristics. OCT excels in the assessment of plaque phenotype and post-procedural complications such as dissections and malapposition [9]. Furthermore, OCT measurements of lumen diameter and area display greater accuracy than those obtained through IVUS and angiography, both in vivo and ex vivo [9]. 

In several pathological investigations, AMI has not been uniformly attributed to plaque rupture (PR); a significant portion of cases arises from alternative pathological mechanisms, including plaque erosion (PE) and calcified nodules (CNs)—phenomena often under-recognized in angiography-guided percutaneous coronary intervention (PCI) and even in (IVUS-guided procedures [22,23]. OCT possesses the capability to classify the underlying etiology of coronary thrombosis into several categories: PR, PE, CNs, spontaneous coronary dissection, and other less common mechanisms [24]. This ability to emulate histopathological examination in vivo presents a fundamental diagnostic advantage. Recent OCT studies have suggested tailored therapeutic strategies that align with specific plaque phenotypes in patients experiencing AMI, thereby potentially enhancing clinical outcomes [25,26,27]. Additionally, OCT plays an essential role in guiding PCI by enabling effective lesion preparation, precise stent sizing and proper stent deployment. It is also critical for verifying adequate stent expansion and apposition, helping to reduce the risks of stent thrombosis and in-stent restenosis. [28,29]. Additionally, OCT offers valuable insights for post-AMI antithrombotic therapy, based on a thorough understanding of PCI results and the underlying mechanisms of acute coronary syndrome (ACS) [21,27]. 

This narrative review highlights the role of OCT in managing AMI, by emphasizing OCT’s dual function as an advanced diagnostic tool and an accurate guide for PCI. It explores how OCT’s detailed imaging capabilities enable a more personalized therapeutic approach, essential in precision medicine. By enhancing tailored approaches, the aim is to optimize therapeutic strategies, thereby improving clinical outcomes for AMI patients. Through this comprehensive analysis, this review depicts OCT as a fundamental technology in modern cardiology.

## 2. Physical Basis of Coronary Optical Coherence Tomography

First introduced in 1991, OCT has revolutionized ophthalmology by providing high-resolution, cross-sectional images of retina and choroid tissues at the micron scale [30]. Over time, OCT has become a fundamental tool in ophthalmic diagnostics, aiding in the evaluation of various conditions, including neovascular age-related macular degeneration (AMD), central serous chorioretinopathy (CSCR), and different retinal vascular disorders. [31]. Its technological advancements have extended its application beyond ophthalmology, finding validation in the cardiovascular domain through both animal [32] and human autopsy models [33].

OCT operates on the principle of low coherence interferometry, which measures the time delay of light scattered from multiple depths within a biological sample. This technique constructs a detailed axial reflectivity profile of the tissue, positioning OCT as a superior imaging modality for evaluating coronary atherosclerotic plaque composition [34]. The resolution of OCT images typically ranges from 10 to 20 μm axially and 20 to 90 μm laterally, with a maximum scan diameter of 6.8 mm [35]. Despite its remarkable imaging capabilities, the penetration depth of OCT is constrained by the optical properties of the tissue, generally ranging between 0.5 and 1.5 mm [34]. Thus, while OCT has emerged as a pivotal imaging technique, its limitations in tissue penetration necessitate ongoing research and development.

OCT utilizes low-coherence interferometry to measure the time delay of light that is back-reflected or back-scattered from different depths within biological tissue, allowing for the reconstruction of the axial reflectivity profile of a sample [36]. Intravascular OCT employs miniature, side-looking fiber optic probes introduced through a narrow, flexible catheter to examine the walls of coronary arteries, creating a helical scanning pattern across the vessel’s luminal surface. However, the strong light scattering and attenuation effects of blood require saline flushing to effectively dilute the blood during imaging. Unfortunately, the volume of saline used and the risk of ischemic complications limit both the duration of imaging and data acquisition [37].

Recent advancements in OCT technology, particularly in the Fourier Domain (FD) and Spectral Domain (SD) configurations, have significantly enhanced imaging capabilities. For instance, FD-OCT can achieve acquisition speeds of up to 100,000 axial lines per second, which allows for the production of 200 frames per second [38]. Conversely, Swept Source OCT (SS-OCT) excels in wavelength-tuning speeds, further improving imaging speed with emerging technologies employing wavelengths greater than 1 μm. SS-OCT’s use of narrow line width swept sources enables superior spectral resolution compared to SD-OCT, making it increasingly preferred for clinical applications [39].

The current leading systems include the OPTISTM, which combines angiographic and OCT imaging, and the Lunawave^®^ system, recognized for its 3-D image reconstructions. Additionally, hybrid catheter systems that integrate OCT and IVUS are being investigated for research purposes, capitalizing on the advantages of both technologies within a single device [40].

## 3. Optical Coherence Tomography (OCT) Findings in Myocardial Infarction

OCT is a valuable diagnostic tool for assessing the culprit lesion in AMI. Although traditional angiography is considered the gold standard for identifying culprit lesions, it can sometimes be challenging when faced with ambiguous angiographic images or multiple potential culprit lesions [41]. Some studies have shown that approximately 30% of patients with non-ST-segment elevation ACS (NSTEACS) [42] and 4–10% of patients with ST-segment elevation MI (STEMI) [43] do not have a clearly identifiable culprit lesion on angiography. In such cases, OCT plays a central role in identifying the culprit lesion, overcoming the limitations of angiography. 

Moreover, OCT also plays a pivotal role in identifying the key characteristics of the culprit lesion and enables the differentiation between the three major histopathologic substrates underlying coronary thrombosis, PR, PE, and CN [21,44], providing in-depth insight into the mechanism of plaque destabilization and the morphological features of the infarct-related segment. This capability allows for a personalized approach in the interventional management of patients with MI, as the revascularization strategy will be guided by the underlying mechanism and the morphological characteristics of the destabilized plaque underlying the ACS. 

### 3.1. Plaque Rupture 

**Figure 1:**
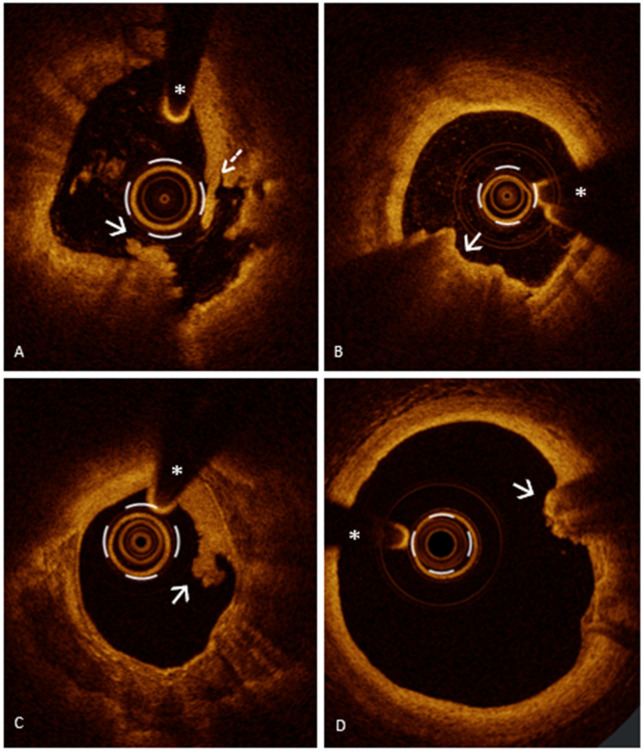
Mechanisms of myocardial infarction: PR and PE. Panel (**A**). PR: rupture of the fibrous cap resulting in a large vessel wall cavity with the exposure of highly thrombotic necrotic core to the blood flow; evidence of mixed thrombus (arrow) and white thrombus (dashed arrow) at both sites of the cavity. Panel (**B**,**C**); definite OCT-erosion: presence of thrombus overlying an intact plaque without discontinuity of the fibrous cap. Panel (**B**): red thrombus at 6–7 o’clock (white arrow) with posterior shadowing, precluding interpretation of the underlying tissue. Panel (**C**): white thrombus at 2–3 o’clock (white arrow) with low backscattering and without posterior shadowing allowing for the interpretation of the underlying plaque phenotype, a very calcified plaque with diffuse calcification (calcium arc of about 270°). Panel (**D**); probable OCT-erosion: presence of red thrombus (high backscattering and posterior shadowing) with attenuation of the underlying plaque (white arrow) without atherosclerotic features proximal or distal to its site. The asterisk is indicating the guide wire artifact.

PR, responsible for about two-thirds of ACS, is the most common lesion phenotype underlying ACS, followed by PE and CN [22,44]. PR (Figure 1, Panel A) is defined as a plaque with a discontinuity of the fibrous cap resulting in the formation of a cavity in the vessel wall and the exposure of the highly thrombotic lipid-rich necrotic core to the blood flow [44]. 

Overhanging thrombi are often observed over the ruptured cap, but their presence is not mandatory for diagnosis of PR, as thrombi might not be present in cases of older PRs due to endogenous thrombolysis or when anti-thrombotic or thrombolytic therapies have been administered before coronary catheterization [21].

**Figure 2:**
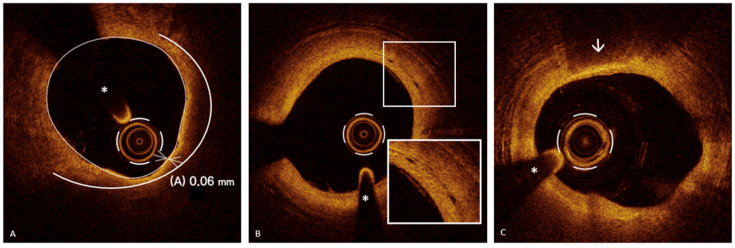
Plaque vulnerability features. Panel (**A**); thin cap fibroatheroma (TCFA): lipid-rich plaque with a lipid arc > 180° (white semicircle) covered by a fibrous cap < 65 µm at its thinnest point (**A**). Panel (**B**); microvessels: round- to oval-shaped structures with black content (“small black holes”) and a diameter of 50–300 μm (magnification in insert). Panel (**C**); macrophages: highly intense backscattering “bright spot”, casting a dark shadow which is sharply bordered laterally. A thin layer of macrophages is observed at 12 o’clock (white arrow). The asterisk is indicating the guide wire artifact.

The mechanisms underlying PR have been extensively investigated, and this ACS mechanism is the only one for which a precursor has been identified: the thin-cap fibroatheroma (TCFA), a plaque characterized by a large lipid core (>180°) covered by a thin fibrous cap (<65 µm) [9], which serves as a prototype for “rupture-prone” plaque [45,46] (Figure 2, Panel A). 

The concept of plaque vulnerability has been extensively investigated, and several studies have concluded that the presence of TCFA, macrophage infiltration (Figure 2, Panel C) (distinct or confluent regions of very intense signal—“bright spots”—generating posterior shadowing [47]), and a small minimum lumen area (MLA) are all characteristics associated with an increased risk of adverse cardiovascular events, defining “rupture-prone” vulnerable plaque [48,49,50,51,52].

Other morphological characteristics associated with plaque vulnerability are neoangiogenesis [53] and cholesterol crystals (CCs) [54,55].

Neoangiogenesis (Figure 2, Panel B) is characterized by the presence of microvessels [53] extending from the adventitia to the intima which allow the influx of lipid material and inflammatory cells into the plaque, promoting its destabilization. By OCT, they are identified as round or oval structures (“small black holes”), signal-poor and sharply delineated, with a diameter of 50–300 μm, and present in at least three consecutive frames [21].

Cholesterol crystals (CCs) are another intraplaque microstructure that may accelerate plaque progression by activating local and systemic inflammation. Due to their pointed shape, CCs can perforate the fibrous cap and contribute to plaque destabilization by increasing local physical stress [54,55,56]. On OCT images, CCs are observed as thin, linear, sharp-bordered regions with bright signal and without posterior shadowing, usually in the context of a lipid plaque [57]. 

In the identification of vulnerable plaques, IVUS also provides important information. This technique allows for the identification of positive vessel remodeling at sites of atheroma formation, measurement of plaque burden, MLA, and recognition of TCFA, all indicators of plaque vulnerability, as demonstrated by numerous studies [58,59,60]. However, IVUS has limitations as it cannot precisely characterize plaque morphology due to its lower spatial resolution. This limitation has been partially overcome by integrating NIRS with IVUS, which provides information about the lipid content of the plaque [61]. Additionally, IVUS does not allow the identification of further vulnerability features such as neoangiogenesis and macrophages, which OCT can detect. In conclusion, both techniques have been employed successfully in identifying vulnerable plaques and in studies correlating plaque vulnerability with adverse cardiovascular events. However, OCT, thanks to its higher spatial resolution, allows for a better characterization of plaque morphology and provides a more complete assessment of vulnerability, as it can also identify additional features such as neoangiogenesis, macrophage infiltration, and CCs.

### 3.2. Plaque Erosion

PE, responsible for about one-third of ACS cases, often found in young women and smokers, is a plaque with endothelial loss or dysfunction in the absence of the rupture of the fibrous cap [44,47,62]. PE is defined by OCT as the presence of thrombus or irregular luminal surface with an intact fibrous cap [21,57]. However, PE remains an exclusion diagnosis in vivo due to the limited resolution of OCT in detecting the endothelial monolayer denudation [21].

A distinction has been proposed between “definite” and “probable” PE: (1) “definite OCT-erosion” is defined as thrombus overlying an intact plaque without evidence of discontinuity of the fibrous cap (Figure 1, Panels B and C); (2) “probable OCT-erosion” is defined by luminal surface irregularity at the culprit lesion without thrombus or evidence of thrombus with attenuation of the underlying plaque without superficial lipid or calcification (atherosclerosis features) immediately proximal or distal to the site of thrombus (Figure 1, Panel D) [57]. While there is a deep understanding of the mechanisms responsible for PR that recognizes TCFA as its precursor, the mechanisms responsible for PE have recently been investigated. Unlike PR, for which a specific underlying plaque morphology has been identified, PE can occur on any substrate and generally involves lesions with more “stable” features [62,63]. Libby et al. identified several processes involved in PE such as high endothelial shear stress, basement membrane breakdown, endothelial cell death and endothelial-to-mesenchymal transition, all leading to impaired endothelial integrity resulting in intracoronary thrombosis [64].

### 3.3. Eruptive Calcified Nodule

**Figure 3:**
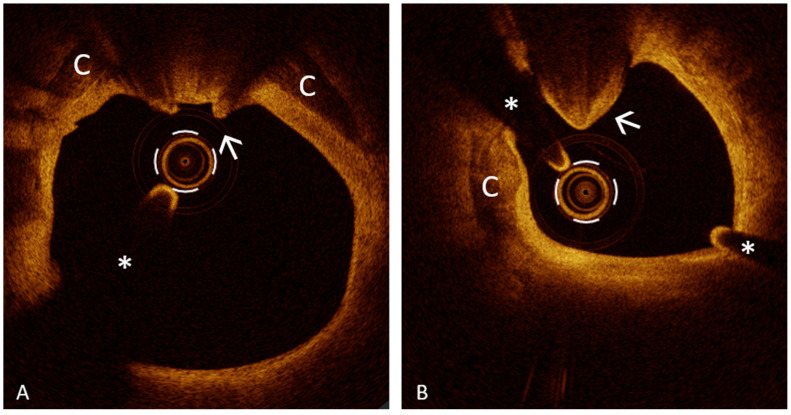
Calcified nodule (CN): calcified lesion protruding into the coronary artery lumen. Panel (**A**): Eruptive CN with fibrous cap discontinuity and overlying mixed thrombus (white arrow) in the context of severely calcified atherosclerotic disease. Spotty calcium is indicated with (C). Panel (**B**); noneruptive CN: Calcified lesion protruding into the vessel lumen without evidence of fibrous cap discontinuity and overlying thrombus (white arrow). Spotty calcium is indicated with (C). The asterisk is indicating the guide wire artifact.

A CN is a calcified lesion protruding into the coronary artery lumen. IVI using IVUS or OCT enables the differentiation of two CN subtypes: noneruptive and eruptive (Figure 2). A noneruptive CN (Figure 3, Panel B) is characterized by a calcified lesion protruding into the vessel lumen with a thick fibrous cap and without overlying thrombus. 

The eruptive CN is a rare cause of ACS, accounting for about 2–7% of culprit lesions [65]. An OCT-eruptive CN (Figure 3, Panel A) features a fibrous cap disruption over a calcified plaque, with protruding, superficial calcium, significant calcium proximal and/or distal to the lesion, and an overlying thrombus [47,57]. Through OCT, eruptive and noneruptive CNs are distinguished from isolated protruding calcium by significant superficial signal attenuation seen in both subtypes [65]. The eruptive CN is often associated with fibrocalcific atherosclerotic plaques. In this context, OCT plays a crucial role not only in identifying this substrate of ACS, but also in the detailed assessment of the calcified segment. OCT provides an extraordinary visualization of calcium, which appears as a low-intensity or heterogeneous structure with sharply defined borders, allowing for the precise measurement of thickness, angular extension, length, and depth (the minimum distance between calcium and the vessel lumen). According to the length and angular extension, calcium is typically distinguished between “spotty calcium” (angular extension < 90° and a length < 4 mm) and “diffuse calcification” [57]. The information provided by OCT regarding CNs and calcified lesions, where these substrates are often found, is crucial for guiding subsequent PCI. In the study by Kondo et al. [66], conducted on 702 ACS patients who underwent OCT-guided PCI, CN accounted for 4% of cases. The 1-year major adverse cardiac events (MACE) rate was notably the highest for patients with CNs at 32.1%, primarily driven by cardiovascular deaths (25.0%). This study indicates that, despite being less common, CNs contribute significantly to adverse outcomes post-PCI, necessitating the further evaluation of OCT-guided interventions based on lesion type [66]. 

### 3.4. Different Culprit Plaque Morphologies Associated with Different Clinical Scenarios

Several OCT studies have shown that PR and PE are actually associated with different clinical manifestations of MI, in contrast with previous pathological studies that suggested a common mechanism underlying both STEMI and NSTEMI. As previously described, PR is the only mechanism of SCA with a well-identified precursor, the TCFA, a prototype of the “rupture-prone” plaque, while PE can occur on any substrate and generally involves stable lesions [62,63]. Several studies have established that PR is more frequent in older, male individuals with multiple cardiovascular risk factors, while PE is more commonly found in younger individuals and female, often smokers, without other cardiovascular risk factors [44,62]. A study conducted by Jia et al. [22], involving 126 patients with ACS undergoing OCT evaluation of the target lesion, showed that NSTEACSs were more common in patients with OCT-erosion and OCT-CN than in those with PR. Moreover, OCT-erosion was characterized by a stable plaque with a thicker fibrous cap and smaller lipid arc compared to PR. An interesting OCT study by Ino et al. [67] investigated the differences of the culprit lesion morphologies between STEMI and NSTEACS, finding a higher prevalence of PR, TCFA, and red thrombus in patients with STEMI compared with those with NSTEACS. In a larger prospective OCT study conducted by Fang et al., 1442 STEMI patients were divided into two groups according to age (≤50 years and >50 years). The study revealed that younger patients (≤50 years) had a higher incidence of PE, a larger MLA, and fewer plaque vulnerability features compared to older patients (>50 years) [68]. Regarding eruptive CNs, several studies have shown that this substrate is more common in calcified arteries, older patients, and males, and is frequently associated with non-occlusive thrombus formation [22]. In line with this evidence, an elegant study was conducted by Weng et al. to examine peripheral atherosclerosis in ACS patients with PR and PE. They found that patients with PR had greater and more vulnerable peripheral atherosclerosis [69]. This study provides a crucial insight into the differences in substrates and clinical manifestations between PR and erosion. PR is more common in older individuals, men, and those with multiple cardiovascular risk factors. In contrast, PE is more frequently observed in younger patients, often women and smokers, but with fewer traditional risk factors. This difference in cardiovascular risk profiles translates into different atherosclerotic phenotypes and, consequently, distinct mechanisms of ACS. This reasoning helps us to understand why patients with PR as a mechanism of ACS are not only suffering from coronary atherosclerosis but also from a systemic and more vulnerable atherosclerotic disease. 

### 3.5. Thrombus

PR, PE, and CN are different mechanisms in pathogenesis, but all lead to the same result: intracoronary thrombosis and consequently ACS. OCT is the gold standard for visualizing intracoronary thrombi, protruding masses attached to the luminal surface or floating within the lumen. OCT allows for the distinction between red thrombus, primarily composed of red blood cells, and white thrombus, composed of platelets, based on the different optical properties of these components. The red thrombus is characterized by an intense signal (“high backscattering”) and produces high signal attenuation with a posterior shadowing, white thrombus has a less intense signal and lower posterior attenuation, and “mixed” thrombus has intermediate features between the previous two [21,47]. It is essential to note that the presence of intracoronary thrombi in patients with myocardial infarction, when assessing the culprit lesion with OCT, may not be detected due to endogenous thrombolysis or the administration of thrombolytic or antithrombotic therapies. Furthermore, the presence of thrombus is necessary for the definitive certain diagnosis of a calcific nodule or “definite OCT-erosion”, but it is not required for confirming PR or for defining a “probable OCT-erosion”. Although it is a rare finding, another OCT observation in patients with MI is recanalized thrombus. This finding is characterized by a “Swiss cheese”, “spider web-like”, “lotus root”, or “honeycomb” appearance, with signal-rich, high-backscattering septa with smooth inner edges, dividing the lumen into multiple small, interconnected cavities [70,71]. Angiography provides only a “lumenography” and may overlook the recanalized thrombus providing an ambiguous angiographic image characterized by “haziness”. In patients with MI where angiography does not reveal a clear culprit lesion, it is crucial to carefully observe areas of “haziness” or “hazy spots” and clarify these ambiguous angiography findings by the use of IVI. 

A semi-quantitative score for intracoronary thrombus using OCT has been proposed: by evaluating the number of quadrants in cross-sectional OCT images, the thrombus is classified as absent (0 quadrants) or as subtending 1 to 4 quadrants. The final score is calculated as the sum of the scores from each cross-sectional image where the thrombus is visible [72]. Additionally, OCT allows for the measurement of thrombus area by outlining the thrombus on cross-sectional images. From this, the thrombus volume can be determined by multiplying the average thrombus area by its length. [73]. In conclusion, OCT is the gold standard for identifying intracoronary thrombi, often presenting with ambiguous angiographic images, for which OCT serves as a clarifier. Furthermore, it can distinguish the morphology and composition of thrombi, differentiating between red, white, and mixed thrombi, and allow for the quantification of the thrombotic burden, guiding subsequent removal treatment [74].

### 3.6. “Healed Plaque” in Myocardial Infarction: Protective Mechanism and Vulnerability Epiphenomenon

**Figure 4:**
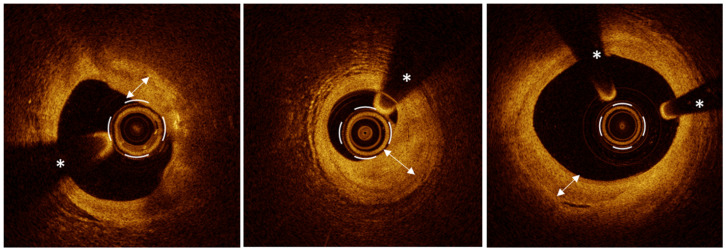
Healed plaque: layered structure with an “onion-like” appearance, featuring one or more layers with intense, heterogeneous signals, layers of different optical densities (double-headed arrow), and a distinct demarcation from the underlying tissue. The asterisk is indicating the guide wire artifact.

Although PR, PE, and CN are involved in the pathogenesis of ACS, plaque destabilization results from a far more intricate interaction between thrombotic factors and healing processes. Recently, the role of plaque “healing” has garnered significant interest due to its potential to promote plaque repair following rupture or erosion, playing a key role in the natural history of atherosclerotic disease and preventing the occurrence of events. Plaque healing consists of three stages: thrombus lysis, granulation tissue formation, and re-re-endothelialization [75]. The “double-hit theory” has recently been proposed, suggesting that the occurrence of an ACS depends on both the destabilization of an atherosclerotic plaque and an ineffective healing mechanism [75]. This process may explain why some high-risk plaques destabilize without leading to ACS, as observed in various pathological studies [76,77]. A “healed” plaque appears on OCT as a layered structure with an “onion-like” appearance, featuring one or more layers with intense, heterogeneous signals and a distinct demarcation from the underlying tissue (Figure 4) [78].

Several studies have investigated “healed” plaques to shed light on the significance of the “healing” mechanism. Vergallo et al. have shown that “healed” plaques are more prevalent in patients with chronic coronary syndrome compared to those with ACS, suggesting its protective role in the onset and recurrence of ACS [79]. Fracassi et al. observed that healed plaques were present at the culprit site in over one-quarter of ACS patients and that these “layered” plaques frequently showed OCT features of vulnerability [80]. In line with these findings, Dai et al. conducted a three-vessel OCT study that investigated the prevalence of healed-culprit and non-culprit plaques in patients with AMI. Specifically, layered plaques were found in three-quarters of patients with AMI, particularly at the culprit sites in patients with STEMI. Patients with layered culprit plaques also had a higher number of layered non-culprit plaques, and these layered plaques showed more severe lumen area stenosis on OCT at both culprit and non-culprit sites compared to the non-layered plaques [81]. These results highlight two aspects of plaque healing: on one hand, its protective role in the occurrence and recurrence of cardiovascular (CV) events, and on the other hand, its significance as an epiphenomenon of vulnerable coronary disease, with layered plaque serving as evidence of previous plaque destabilization [75,79,80,81]. Additionally, this phenomenon is associated with a progressive reduction in MLA, which is a well-established predictor of CV events [48]. Future studies are needed to determine whether plaque healing should be recognized as a protective mechanism in its own right or if it should only be considered as a marker of vulnerability. Specifically, we need to assess whether healing represents an effective repair process or if it indicates a propensity for plaque destabilization, where this vulnerability might outweigh the plaque’s ability to self-repair after rupture or erosion. 

### 3.7. The Role of OCT in Myocardial Infarction with Non-Obstructive Coronary Arteries (MINOCA)

Recently, OCT has demonstrated significant value in the diagnostic evaluation of myocardial infarction with non-obstructive coronary arteries (MINOCA). MINOCA refers to MI with mild (<50% diameter stenosis) or no obstructive coronary artery disease (CAD) on angiogram. This condition accounts for 6–15% of spontaneous MIs and is often found in women and in patients without common CV risk factors [82]. Among the most common causes of MINOCA are the destabilization of atherosclerotic plaques (including PR, PE, and calcific nodules) not causing occlusive or subocclusive intracoronary thrombosis, coronary embolization, spontaneous coronary artery dissection (SCAD), and spasm of an epicardial coronary artery or coronary microvascular dysfunction. In these cases, angiography can often be silent, and the use of intravascular imaging plays a crucial role in clarifying the diagnosis. It is important to note that there are also conditions that can mimic MINOCA, such as non-ischemic cardiomyopathies and myocarditis, and cardiac magnetic resonance (CMR) imaging is particularly useful for differentiating them from MINOCA [82]. Several IVUS and OCT studies showed multiple plaque mechanisms underlying MINOCA such as PR, erosion, vasospasm, embolization or SCAD, which are often not apparent on angiography [83,84,85,86]. 

SCAD is a condition characterized by the spontaneous, non-iatrogenic and non-traumatic separation of the layers of the coronary artery wall. This separation allows blood to accumulate within the medial space, compromising the coronary circulation and ultimately leading to myocardial ischemia and ACS [87]. While the precise causes of SCAD are not fully understood, several well-documented risk factors have been identified, including extreme physical exertion, pregnancy or the postpartum period, fibromuscular dysplasia, connective tissue disorders, and lifestyle factors like cigarette smoking [88]. The pathogenesis of SCAD involves a tear or disruption in the coronary artery. There are two primary mechanisms proposed to explain how SCAD develops: the “inside-out phenomenon” and the “Outside-In Phenomenon”. The “Inside-Out phenomenon” refers to a mechanism in which a tear occurs in the inner layer of the coronary artery and blood leaks into the middle layer creating a “false” lumen. The angiographic features of this phenomenon include contrast dye staining the arterial wall and the presence of multiple radiolucent lumens [88]. The “outside-in phenomenon” refers to the accumulation of blood within the arterial wall due to the rupture of small blood vessels within the media, without a tear occurring in the intima. The resulting intramural hematoma compresses the artery from the outside, causing narrowing that can mimic atherosclerotic stenosis [88]. Saw proposed an angiographic classification for SCAD, identifying four distinct angiographic subtypes. Type 1 is characterized by contrast dye staining the arterial wall and the presence of multiple radiolucent lumens (29–48% of cases); type 2, the most common (60–75% of cases), is characterized by diffuse smooth stenosis and can be further divided into two subtypes according to the extent of the dissection to the terminal segment; type 3 SCAD (2–4% of cases) describes a focal area that appears similar to an “atherosclerotic” lesion with hazy stenosis and linear and long lesions (11–20 mm); and type 4 SCAD, typically involving a distal segment of the artery, is marked by total coronary occlusion [87,88]. Traditional angiography often leads to under-diagnosis, particularly in some SCAD type 2 cases and in most type 3 and 4, where the use of IVI with IVUS or OCT plays a pivotal role in the diagnosis. Compared to IVUS, OCT offers more precise diagnostic information regarding intramural hematomas and intimal tears. However, utilizing OCT in a dissected vessel carries risks, such as the potential for worsening the dissection or causing vessel closure due to the injection of contrast media. Therefore, OCT should be reserved for cases with a high clinical suspicion, where angiography alone leaves diagnostic uncertainty. The primary diagnostic OCT feature of SCAD is a dissection flap, visible in otherwise normal arterial segments without atherosclerosis. OCT, with its high resolution, significantly enhances the detection of intimal flaps and can identify rupture sites not visible on angiography or missed by IVUS. Additionally, another key diagnostic element of SCAD is the intramural hematoma. Two primary pathogenic mechanisms have been previously described for intramural hematoma formation: an intimal tear that creates a double lumen and disrupts blood flow, and a spontaneous hematoma in the media that can cause lumen narrowing without the typical double lumen appearance. Although the presence of an intimal flap can confirm the diagnosis, it is not always present. Therefore, the presence of an intramural hematoma alone is highly significant for diagnosis, making it sufficient for confirming SCAD even in the absence of an intimal flap.

In the context of MINOCA, OCT emerges as a pivotal diagnostic tool, effectively addressing the shortcomings often encountered with conventional angiography. Traditional angiographic techniques frequently fall short in delineating a clear culprit lesion, particularly in cases characterized by subtle or non-obstructive pathology. In such instances, OCT offers significant advantages by providing high-resolution imaging that facilitates the identification of distinct underlying mechanisms responsible for the AMI.

Specifically, OCT can elucidate critical conditions such as PR or erosion that may not result in obstructive thrombosis. Furthermore, it is adept at detecting other complex scenarios like SCAD and coronary embolization—pathological states where standard angiographic methods may yield ambiguous results or completely inconclusive images. By offering an enhanced visualization of the coronary artery structure and detailed characterization of vascular lesions, OCT plays a crucial role in refining the diagnostic process. Consequently, this advanced imaging modality not only aids in achieving a precise diagnosis but also contributes to informing appropriate therapeutic strategies, ultimately enhancing patient care for those affected by MINOCA. Thus, OCT represents an invaluable asset in the diagnostic toolkit for clinicians confronting these intricate and challenging CV conditions.

### 3.8. The Concept of “Pancoronary” Vulnerability and Non-Culprit Lesions

Several studies have examined the non-culprit lesion (NCL) phenotype in patients with ACS. The PROSPECT (Providing Regional Observations to Study Predictors of Events in the Coronary Tree) study investigated the frequency of NC PR in 697 patients with ACS performing ultrasound virtual histology (IVUS-VH) of all three coronary arteries [89]. This study demonstrated that NC PRs occurred in 14% of patients with ACS. It found that the plaque burden was significantly greater in NC lesions with a rupture compared to those without. Additionally, NC lesions with a rupture were more frequently classified as fibroatheromas. However, over a 3-year follow-up, the overall incidence of major adverse cardiac events did not significantly differ between patients with and without NC PRs [89]. In light of the systemic nature of atherosclerotic disease, an in vivo three-vessel OCT study conducted by Vergallo R. et al. investigated the morphological characteristics of NC plaques in patients with and without PR in culprit lesions [90]. The study revealed that patients with PR in culprit lesions exhibited a higher prevalence of thin-cap TCFAs in NCLs, demonstrating the existence of a “vulnerable” phenotype of atherosclerotic disease [90]. Additionally, an in vivo three-vessel OCT study investigating NCLs demonstrated that patients with NC PR exhibited a pancoronary vulnerable phenotype characterized by greater evidence of TCFA, neovascularization, and macrophage infiltration compared to patients without NC PR [91]. These studies have emphasized the systemic nature of atherosclerotic disease, thereby extending the concept of a “vulnerable lesion” to that of “vulnerable atherosclerotic disease” [90,91]. Several intravascular imaging studies have demonstrated the association between vulnerable plaques and the occurrence of major adverse CV events (MACE). In this regard, four major prospective studies aimed to identify, through IVI, patients at high risk of future events who may benefit from specific preventive medical therapy. The ATHEROREMO-IVUS study, which included 581 patients undergoing coronary angiography and IVUS, found that TCFA and a plaque burden >70% were associated with a higher incidence of MACE at one year [92]. Subsequent studies using NIRS-IVUS, the Lipid Rich Plaque Study [93], and the ATHEROREMO-NIRS sub-study [94], demonstrated that greater lipid content in plaques was associated with worse clinical outcomes. Finally, the CLIMA study, which employed OCT and enrolled 1003 patients, concluded that patients with TCFA lesions, large lipid arcs (>180°), and macrophage accumulation had a worse prognosis in terms of CV death and target vessel-related myocardial infarction compared to patients with plaques without these vulnerability features [48]. 

These studies have highlighted that NCLs can evolve into future culprit lesions, becoming responsible for recurrent ACS. This raises the issue of the need for complete revascularization in patients with ACS with multivessel disease (MVD). The current ESC Guidelines for the management of ACS [12] provide different recommendations based on the clinical presentation, particularly in the presence or absence of cardiogenic shock. For patients with hemodynamically stable STEMI and MVD undergoing primary PCI (pPCI), complete revascularization is recommended either during the initial PCI procedure or within 45 days, with PCI of non-infarct-related lesions guided by angiographic severity (Class I, Level of Evidence B). For patients with hemodynamically stable NSTEACS, complete revascularization should be considered, ideally during the initial procedure (Class IIa, Level of Evidence C). While the invasive epicardial functional assessment of NC segments of the IRA is not recommended during the initial procedure (Class III, Level of Evidence C) in STEMI patients, it may be considered (Class IIb, Level of Evidence B) in NSTEACS patients [12]. Several studies have attempted to determine the best strategy for guiding complete revascularization by comparing different strategies, such as stress echocardiography [95] or Fractional Flow Reserve (FFR) versus angiography [28,96], without demonstrating differences in the recurrence of CV events. Currently, no superior guidance strategy has been established, highlighting the need for large trials to compare these strategies and explore the roles of intravascular imaging guidance in the revascularization of NCLs.

Given the demonstrated link between vulnerable plaques and MACE, recent trials have investigated whether a “preventive” stenting strategy could reduce MACE and enhance clinical outcomes. 

The PROSPECT ABSORB trial by Stone et al. [97] assessed the outcomes of PCI for non-flow-limiting vulnerable plaques. In this study, 898 patients with MI underwent PCI for all flow-limiting lesions. Subsequent imaging of the three vessels using IVUS-NIRS was conducted, and patients with angiographically non-obstructive stenosis but an IVUS plaque burden of ≥65% were randomly assigned to receive either a bioresorbable vascular scaffold (BVS) combined with guideline-directed medical therapy (GDMT) or GDMT alone. At the 25-month follow-up, lesions treated with BVS demonstrated more than double the minimal lumen area (MLA) compared to those receiving only GDMT. Although the study was not sufficiently powered to evaluate clinical outcomes, there was a trend indicating fewer MACEs in the BVS group compared to the GDMT-only group, largely due to a reduction in cases of progressive angina that required revascularization [97]. Similarly, the PECTUS trial [98] aimed to determine whether preemptive stenting of OCT- identified vulnerable plaques using Absorb BVS, combined with guideline-directed medical therapy (GDMT), could reduce MACEs compared to GDMT alone. However, the trial was prematurely halted due to the withdrawal of Absorb BVS from the market, and thus, no significant results were obtained [98]. The recently published PREVENT trial is the largest study to address this issue [99]. It aimed to determine whether preventive PCI of non-flow-limiting vulnerable plaques leads to better clinical outcomes compared to optimal medical therapy alone. The trial enrolled 1606 patients with ACS or chronic coronary syndrome (CCS) with non-flow-limiting vulnerable plaques identified through IVUS, NIRS, or OCT. Participants were randomly assigned to either PCI plus GDMT or GDMT alone. The results demonstrated that preventive PCI of non-flow-limiting vulnerable plaques significantly reduced MACEs compared to GDMT alone [99].

OCT has emerged as a useful tool in assessing NCLs in patients with MI. It plays a significant role as guidance for PCI when NCLs are obstructive and flow-limiting, thus supporting complete revascularization. Additionally, OCT is useful for evaluating NCL phenotypes and vulnerability, potentially guiding the treatment of lesions that, while not obstructive or flow-limiting, are still highly unstable. Additionally, OCT provides valuable insights into the overall disease phenotype of a patient. By characterizing the extent and nature of coronary artery disease, OCT helps in devising personalized secondary prevention strategies for patients at high risk. This approach aligns with the concept of pancoronary vulnerability, which emphasizes the importance of addressing not just the culprit or obstructive, flow-limiting lesions but also the broader spectrum of coronary disease that might contribute to future adverse events. 

### 3.9. Stent Thrombosis as Cause of MI 

The advent of drug-eluting stents (DES) with progressive technological improvements in the stent platform, the polymer coating, and the antiproliferative agents released from the stent, combined with the progress in PCI techniques and antithrombotic treatments, have significantly lowered the incidence of in-stent restenosis (ISR) and stent thrombosis (ST). ST is a rare but catastrophic complication and currently accounts for up to 20% of MI events after PCI [100].

The Academic Research Consortium (ARC) has classified ST based on the degree of diagnostic certainty and the timing of occurrence relative to the index procedure [101]. 

ST is categorized based on the level of certainty into definite, probable, and possible classifications. Definite ST requires angiographic or postmortem evidence of thrombotic stent occlusion, probable ST refers to any unexplained death within 30 days of stent implantation or any MI related to the previous stented area, and possible ST refers to any unexplained death occurring more than 30 days after stent implantation [100,101]. Depending on when it occurs in relation to the index procedure, ST can be categorized as early (which includes acute—within 24 h—and sub-acute—within 1 to 30 days), late (occurring between 30 days and 1 year), and very late (more than 1 year after stent implantation) [100,101]. Several risk factors for ST have been identified, related to the patient, the lesion, the stent, and the procedure. The patient-related risk factors for early ST include the early discontinuation of dual antiplatelet therapy (DAPT), an acute clinical presentation, genetic variants, and a reduced left ventricular ejection fraction. For late and very late ST, risk factors include diabetes mellitus (DM), chronic kidney disease (CKD), younger age, a history of malignancy, and peripheral artery disease. Active smoking is a common clinical risk factor for both acute and late ST. Among lesion-related risk factors for early stent thrombosis are lesions in the left main coronary artery, TIMI flow grade < 3, small vessel CAD, complex lesions, and severe restenosis, while thrombus and bypass graft lesions are more often related to late or very late ST [100]. Left anterior descending artery (LAD) lesions, bifurcation lesions, and severely calcified lesions are associated with both early and late ST. Stent-related risk factors include stent strut thickness and small stent diameter for early ST, stent number for late or very late ST, and stent length for both. Risk factors that can be most effectively managed to limit ST are primarily related to the procedure itself. Among procedure-related risk factors are stent undersizing, underexpansion and malapposition, edge dissection, stent fracture, and residual stenosis for early ST, and persistent uncovered struts, late strut malapposition, stent overlapping, and neoatherosclerosis as causes of late ST [100]. OCT is an invaluable diagnostic tool in this scenario. It identifies procedure-related risk factors during the main procedure and allows for their correction, thereby playing a preventive role in ST. Moreover, OCT is crucial for assessing the underlying mechanism of ST and guiding treatment, providing detailed information that helps tailor therapeutic strategies based on the specific issues identified.

#### 3.9.1. Mechanisms of Stent Failure Underlying ST

As previously described, various mechanisms of stent failure can lead to ST, a rare but catastrophic complication accounting for up to 20% of MI events after PCI [64]. Early ST is typically caused by stent undersizing, underexpansion, acute stent malapposition, edge dissection, and stent fracture. In contrast, late and very late ST are commonly associated with persistent uncovered struts, late malapposition, and neoatherosclerosis. In this context, OCT stands out as the most informative tool. It provides critical insights into these mechanisms, helping to identify the underlying causes of ST and to guide the selection of appropriate treatment strategies [102].

Stent expansion refers to the minimum stent area (MSA), measured either as an absolute value (absolute expansion) or relative to predefined reference areas. Small MSA is known as a well-established predictor of suboptimal post-PCI fractional flow reserve (FFR) values, as shown in the DOCTORS trial [102], and data from the CLI-OPCI registries suggest that an MSA of 4.5 mm^2^ can discriminate patients at risk of MACEs [103]. For left main (LM) lesions, higher cut-off values are required, such as >7 mm^2^ for distal LM and >8 mm^2^ for proximal LM, as measured by IVUS [104]. The EAPCI document recommended to achieve a relative expansion > 80% as the goal of PCI optimization [104], according to the DOCTORS Study, in which the optimal cut-off value of stent expansion able to predict FFR > 0.90 was >79.4% [105]. 

**Figure 5:**
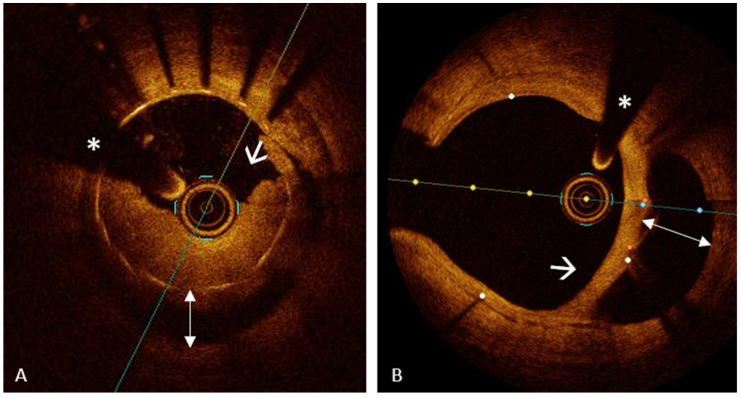
Stent malapposition: separation of at least one stent strut from the intimal surface of the coronary artery wall with a distance between the strut’s surface to the luminal surface greater than the strut thickness. Panel (**A**): in-stent thrombosis with a mixed thrombus (white arrow) associated with the major stent malapposition (double-head arrow) of the previous implanted stent. Panel (**B**): major stent malapposition (double-head arrow) with neointimal hyperplasia (white arrow) embedding the stent struts (resulting in a “dual” lumen appearance). The asterisk is indicating the guide wire artifact.

Stent malapposition (SM) is defined as the separation of at least one stent strut from the intimal surface of the coronary artery wall without the involvement of the side branches with a distance between the strut’s surface to the luminal surface greater than the strut thickness (Figure 5) [104]. Acute SM (ASM) is identified during the index procedure, whereas late SM (LSM) is observed during follow-up. LSM can be further divided into late persistent SM (LPSM), which is an ASM that continues to be present at follow-up, and late acquired SM (LASM), which is detected at follow-up but was not present during the index procedure [104]. ASM is most frequently due to an undersized stent or an ectasia of the vessel. Several studies have reported that 50% of ASM resolved at follow-up [106], with the distance between the stent strut and the vessel wall correlating inversely with the resolution. LPSM is an ASM that has not resolved and is present at follow-up, while LASM is most frequently due to the subsequent mechanisms of positive vessel remodeling, in most cases related to chronic inflammation. Obviously, to distinguish LPSM from LASM, it is necessary to perform IVUS or OCT at the index procedure and at follow-up. Regarding clinical outcomes, several OCT and IVUS studies [106,107] consistently showed that ASM did not affect prognosis, whereas studies on LSM showed conflicting results [106,108,109,110]. Anyhow, other OCT studies and case reports [111,112,113,114] showed that SM (ASM and LSM) is a common OCT finding in patients with acute, subacute, late, and very late ST. A recent study from Kim et al. [115] showed that patients with significant SM after PCI with DES (total malapposition volume, TMV >= 7.0 mm^3^) had a higher rate of major safety events (MSE) including cardiac death, ST, and target vessel-related MI, and that this grade of malapposition correlates with a higher risk of LSM at follow-up. This finding could explain the correlation with long-term MSE [115]. 

Therefore, it is clear that further studies are needed to understand the clinical outcome of SM. Despite the current uncertainties, the European Association of Percutaneous Cardiovascular Intervention (EAPCI) recommend treating ASM > 0.4 mm with longitudinal extension > 1 mm (below these cut-offs, ASM is more likely to spontaneously resolve) [104].

OCT plays a crucial role in identifying stent edge dissection (SED), as it allows for the detection of even subtle stent edge dissections with significantly higher sensitivity compared to IVUS [21]. According to the ILUMIEN III [116], stent edge dissections can be classified as major or minor based on their extent. Major edge dissections are those that extend ≥60 degrees from the vessel circumference or are ≥3 mm in length. Dissections that do not meet these criteria are classified as minor. Data from the CLI-OPCI II [103] and HORIZON-AMI [117] studies revealed that major stent edge dissection (disruption of the vessel media, angular extension > 60°, and length > 2 mm) are those with the highest risk of ST. In particular, in the CLI-OPCI II study, only distal edge dissection with a width ≥ 2 mm (not proximal) was an independent predictor of adverse events [103]. The current EAPCI document recognized the presence of residual plaque burden, extensive lateral (>60°), longitudinal extension (>2 mm), involvement of media or adventitia, and distal localization as established factors increasing the risk for adverse events. OCT plays a pivotal role not only in identifying SED but also in helping to avoid it. To mitigate the risk of SED, it is important to carefully assess the target segment to avoid oversizing and the stent edge landing in atherosclerotic areas and perform careful postdilation, even with a downsized balloon, to minimize the risk of affecting unprotected plaque areas. 

According to the PRESTIGE registry [111], uncovered stent struts are responsible for 64% of early ST and 20% of very late stent thrombosis, while PESTO [112] registries attributed 11% of very late ST to uncovered stent struts. After stent implantation, a substantial number of struts may initially remain uncovered, increasing the risk of early ST. However, as neointimal growth and re-re-endothelialization progress, the number of uncovered struts gradually declines. Persistent uncovered struts, particularly after discontinuation of DAPT, are more commonly associated with late and very late ST [104]. Currently, the definition of strut coverage by OCT is the presence of tissue overlying stent struts > 0 µm [118]. The percentage of uncovered struts can be calculated by dividing the number of uncovered struts by the total number of analyzable struts and then multiplying by 100 [21]. 

#### 3.9.2. Neoatherosclerosis

**Figure 6:**
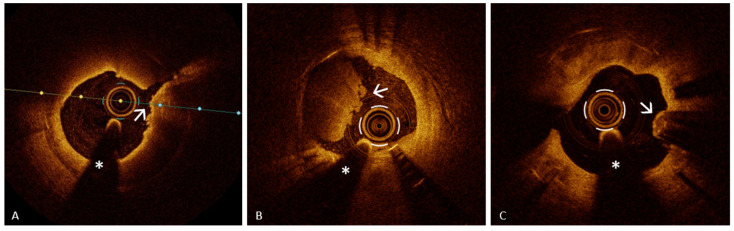
Neoatherosclerosis: the presence of at least one component of a mature atherosclerotic plaque, such as lipid-rich tissue or calcification, within the neointima. Panel (**A**): PR (with arrow) with overlying microthrombi in neoatherosclerosis with unstable plaque characteristics (lipid-laden neointima with a thin fibrous cap). Panel (**B**): definite OCT-erosion with mixed thrombus (with arrow) in fibrocalcific neoatherosclerosis. Panel (**C**): eruptive CN with irregular surface (with arrow) in neoatherosclerosis with diffuse calcification. The asterisk is indicating the guide wire artifact.

Neoatherosclerosis is one of the major substrates of late ST [111,112]. It is pathologically characterized by lipid or calcifications within neointima with a broad phenotypic spectrum, similar to native atherosclerosis. Neointimal formation is a normal healing response in vascular tissue after stent implantation, triggered by the interaction of a platelet-fibrin thrombus with smooth muscle cells and followed by extracellular matrix secretion. However, this physiological reactive mechanism can result in an excessive neointima formation, which can cause ISR. OCT is a valuable tool to assess late stent changes and can identify three different neointima patterns, homogeneous, heterogeneous, and layered, enabling a tailored strategy to treat ISR [21]. Unlike neointima, which is a physiological healing process following stent implantation, neoatherosclerosis is considered its pathological progression. The OCT definition of neoatherosclerosis is the presence of lipids or calcium within the neointima with a longitudinal extension ≥ 1 mm [119]. The OCT appearance of lipids and calcium in neoatherosclerosis is similar to that observed in native vessel atherosclerosis. Likewise, in neoatherosclerosis, macrophages and microvessels can be identified just as they are in native vessels. Furthermore, neoatherosclerosis can undergo the same destabilization mechanisms as native atherosclerosis, potentially causing very late stent thrombosis (Figure 6) [119]. Several studies have explored the correlation between native atherosclerosis and neoatherosclerosis. One mechanism linking these two pathological processes is inflammation: inflammatory cytokines in the native plaque can attract inflammatory cells and growth factors, promoting the development of neoatherosclerosis. Another mechanism involves lipid plaques targeted by stenting: the lipid core, embedding stent struts, can inhibit drug release from the DES, delaying strut coverage and resulting in ineffective healing that promotes the development of neoatherosclerosis. Additionally, stent implantation alters the hemodynamic conditions of the treated segment and is comparable to the endothelial shear stress that causes atherosclerosis in native vessels [119].

### 3.10. Artificial Intelligence (AI) and OCT

The use of artificial intelligence (AI) in OCT is becoming increasingly important, especially in medical fields like CV and ophthalmological diagnostics. By integrating machine learning (ML) and deep learning (DL) techniques with OCT data, the effectiveness and precision of these analyses have significantly improved [120]. In the field of ophthalmology, OCT is a standard tool for diagnosing and monitoring retinal and corneal diseases such as AMD, glaucoma, and diabetic retinopathy. Research in ophthalmology has advanced further than in the CV field regarding AI integration with OCT. DL algorithms have been trained to automatically detect these conditions with high accuracy, providing real-time insights that can aid ophthalmologists in diagnosing and monitoring disease progression [121]. As of today, the Ultreon™ 2.0 Software from Abbott is the only AI-certified software 2.0 for CV OCT. While it represents a solid starting point, especially as a tool that can guide PCI, we are still far from having a system capable of providing a detailed morphological analysis of the plaque. Ultreon™ 2.0 offers fast and efficient decision-making for PCI through AI-enabled calcium detection and EEL measurements, which support vessel preparation strategies. It allows clinicians to quickly determine optimal stent sizing and plan for precise stent placement [122]. Additionally, it is the only platform offering co-registration, with enhanced precision through Dynamic Angio, featuring zoomable, side-by-side live angiography synced with OCT to assist users in accurately guiding stent deployment [122]. In conclusion, AI is revolutionizing the use of OCT, particularly in ophthalmology, where the research is more advanced compared to the CV field. AI, reducing the time required for clinicians to analyze a large volume of OCT scans, might provide faster and more efficient care. However, AI will always fall short when compared to human intelligence, particularly in correlating visual data from OCT with the broader clinical reality. A clinician’s ability to interpret an image in the context of a patient’s medical history, symptoms, and individual characteristics remains irreplaceable. Human intelligence, with its nuanced understanding of clinical conditions, integrates visual information with real-world patient variables, offering a level of contextualization and personalization that AI alone cannot achieve. Therefore, while AI can significantly enhance diagnostic tools, it will always function best as an assistant to, rather than a replacement for, human expertise.

## 4. OCT-Guided P-PCI

### 4.1. Potentiality of OCT in P-PCI

Intracoronary imaging techniques have rapidly evolved over recent decades, addressing observer bias and interobserver variability in the subjective interpretation of coronary angiography. OCT has become increasingly utilized in guiding coronary procedures, and also in the context of ACS, due to its ability to identify ACS mechanisms and detect thrombi. Its high sensitivity currently positions it as the gold standard among imaging techniques. Furthermore, OCT enables the differentiation of pathophysiological mechanisms responsible for ischemia, such as PR, erosion, nodular calcifications, spontaneous coronary dissections, and hematomas. It also facilitates plaque characterization (fibrotic, calcified, or lipid-rich), measurement of cap thickness (an indicator of plaque vulnerability), and identification of macrophage aggregates and microvessels. The lipid content of the plaques is directly proportional to the no-reflow phenomena and reduced blood flow following stent implantation [123]. Consequently, in ACS cases characterized by a higher atherothrombotic burden and plaque fragility, an initial OCT evaluation supports safer stent implantation, reducing complications such as distal embolization. Post-implantation assessments can help quantify intrastent plaque protrusion—an independent predictor of outcomes—and allow for the necessary corrections [124]. Additionally, OCT can identify lipid-rich plaques near the stent edges, particularly those with circumferential extensions greater than 185° and a residual plaque burden exceeding 50%, both of which are associated with increased MACE at one year and a higher risk of in-stent restenosis (ISR) [103,125]. According to the 2023 European Society of Cardiology (ESC) guidelines on ACS [12], intracoronary imaging is recommended for treating evident culprit lesions suitable for PCI (Class IIa evidence) and in ACS cases without significant obstructive coronary artery disease on angiography (e.g., MINOCA). The exclusion of atherothrombotic causes can offer critical support in invasive management and antithrombotic therapy. It is also useful where the culprit lesion is unclear (seen in up to 30% of patients with suspected NSTEMI) or when there are multiple lesions (Class IIb evidence) [42,61]. The culprit lesion is typically indicated by changes in the ECG or by the presence of thrombotic or hazy regions on coronary angiography, though identification can be challenging, especially in multivessel disease. Moreover, 15% of patients undergoing primary PCI (P-PCI) present with a patent vessel with TIMI 3 flow on angiography [126]. In such cases, as noted earlier, OCT can provide precise information on the pathophysiological substrate responsible for the ACS, such as identifying ruptured plaques or assessing plaque composition. OCT is also recommended in cases of left main stenosis, bifurcations (especially those requiring two stents), and complex coronary lesions where achieving an optimal luminographic result is uncertain [127]. OCT provides crucial advantages in lesion preparation, guiding the selection of stent size and length based on lesion severity and MLA. It also detects acute complications such as stent edge dissections, distal embolization, underexpansion, and stent malapposition, as well as mechanisms of stent failure, making OCT invaluable not only for accurate diagnosis but also for determining therapeutic strategies. Multiple studies have evaluated the efficacy and feasibility of OCT-guided PCI, including in ACS treatment, demonstrating that OCT can significantly influence operator decisions and modify revascularization strategies, potentially avoiding unnecessary procedures. 

### 4.2. Trials on OCT-Guided P-PCI Strategies

In 2015, Souteyrand G. et al. [128] published an observational study of invasive treatment without stent implantation in 852 ACS patients with high thrombotic burden, where treatment decisions were guided by OCT. The study demonstrated that conservative management without stent implantation was safe, due to OCT’s sensitivity in identifying the culprit lesion characteristics overlooked by angiography. At 12 months, only one non-fatal myocardial infarction and one PCI for angina were recorded. In a randomized study by Kala et al. [129], 201 suspected STEMI patients underwent angiography-guided or OCT-guided P-PCI. OCT-guided optimization occurred in 29% of cases, addressing issues such as malapposition (59%) and dissections (41%). After 9 months, the OCT group showed significantly less in-segment stenosis compared to the angiography group. The DOCTORS trial [105], which randomized 240 NSTEMI patients to OCT- or angiography-guided PCI, revealed that OCT improved post-PCI FFR values. Stent optimization was achieved in half of the OCT-guided cases compared to 22.5% in the angiography group, with stent expansion increasing from 78.9% to 84.1%. No significant differences in clinical outcomes were observed between the two groups after 6 months. In the EROSION study [130], OCT identified PE and guided treatment with thromboaspiration and/or glycoprotein IIb/IIIa inhibitors followed by antiplatelet therapy, avoiding stent implantation in cases of residual stenosis below 70%. This approach significantly reduced the thrombus volume in 78% of patients within one month, and 92.5% remained event-free after one year [130]. In 2022, EROSION III [27] was published, aiming to verify OCT’s ability to provide additional useful data compared to angiography and whether this information would lead to a change in reperfusion strategy and improve outcomes in 246 patients with STEMI and early patency of the infarcted coronary artery. In this study, OCT-guided P-PCIs resulted in fewer stents being implanted (15% less compared to the angio-guided group) and better residual stenosis results on angiography in stented patients (8.7% OCT-guided vs. 11.8% angio-guided), suggesting the value of OCT imaging in optimizing reperfusion strategies in STEMI patients [27]. A recent meta-analysis by Stone et al. [131] was published with the goal of providing data supporting the better clinical outcomes of patients undergoing OCT- or IVUS-guided PCI in terms of all-cause death or MI compared to angiography-guided PCI. This aimed to fill the gap in data, as previous meta-analyses had only addressed the reduction in composite adverse cardiac events and repeat revascularizations with intracoronary imaging [131]. The number of enrolled patients was 15,964 across 22 trials. Among these, patients with ACS who underwent OCT imaging were extracted from trials such as those by Kim et al. [132], OCTACS [133], DOCTORS [105], ROBUST [129], ILUMIEN III [116], iSIGHT [134], ILUMIEN IV [135], OCTOBER [136], and OCTIVUS [137]. The average follow-up period was 24 ± 7 months. OCT (and IVUS)-guided PCIs were shown to be safer and more effective, with a reduced risk of death, MI, repeat revascularization, and IRS compared to angiography-guided PCIs. Furthermore, when compared to IVUS, OCT demonstrated superior resolution and greater accuracy in plaque characterization as well as lesion measurement. As a result, this meta-analysis provided robust, numerous, and convincing data on the benefits of using intracoronary imaging during PCI, even in the ACS setting, improving both long-term safety and treatment efficacy. To address the limited availability of data on the OCT-guided treatment of complex lesions in ACS, a multicenter Indian registry was published in 2023, which included 500 patients (22% of whom presented with STEMI) [138]. The primary endpoints were to evaluate MLA and the incidence of acute kidney injury (AKI) due to contrast-induced nephropathy (CIN) following OCT-guided PCI. The secondary objectives included evaluating the rates of MACE, cardiac death, and MI at 30 days, 6 months, and 1 year. A notable shift in strategy after OCT was identified in 65% of the lesions (which comprised a pre-procedural adjustment in 52% and a post-procedural adjustment in 30%). Strategy modifications following OCT were seen in 63% of small vessel lesions and 66% of large vessel lesions. Post-procedural optimization occurred in 32% of small vessel lesions compared to 29% in larger vessels. This study, therefore, emphasizes the concept that in complex cases, OCT guidance is safe and can help optimize stent expansion and detect edge dissections, not only in controlled trials but also in clinical practice, particularly in ACS patients. An unusual case in which OCT proved to be particularly useful was presented in a study by Lee T. et al. [139], which recruited 889 patients (48% ACS). Among those with ACS, 4.2% had a calcium nodule, most frequently located in the ostial or mid-right coronary artery as the culprit lesion. Finally, in the case of SCAD, no randomized controlled trials (RCTs) have currently been conducted on the use of OCT for such complications. However, some cohort studies and expert opinions provide guidance [140,141]. In general, there are certain cases where the use of intracoronary imaging can be decisive and thus justified, but it is still necessary to consider certain risks related to vessel tortuosity, diameter, and the distal location of the lesions. 

### 4.3. Limitations and Critical Issues

Despite the notable benefits mentioned, OCT presents some limitations, such as limited tissue penetration (1–2 mm) and signal attenuation due to the presence of red thrombus, lipid core, or necrotic core. Additionally, its use is restricted by aorto-ostial lesions [142], as well as large-caliber or highly tortuous vessels, as these may prevent perfectly circumferential imaging due to the incorrect non-central position of the OCT catheter within the vessel. Moreover, because OCT requires temporary blood removal and flushing with contrast media before imaging, it should be used with particular caution in patients with significantly impaired renal function or in those with severely compromised left ventricular function or hemodynamic instability, as well as in patients with a single remaining vessel. These limitations, which are quite frequent in ACS, had already been analyzed in a 2008 study by Yamaguchi et al. [143], where 76 patients were evaluated using the occlusive technique to assess the safety and feasibility of time-domain OCT, the first-generation OCT. The most common intraprocedural complications were transient events such as ST-T segment changes on ECG, chest pain, bradycardia or tachycardia, but no major life-threatening complications occurred, nor were there acute events such as acute vessel occlusion, dissection, thromboembolism, or vasospasm. The risk of such events has been significantly reduced with the introduction of the non-occlusive technique and, subsequently, FD-OCT, where the high pullback speed allows data acquisition in just a few seconds, without inducing significant ischemia. The safety of FD-OCT has also been evaluated in several studies, including the one by Imola et al. [144], which concluded without any instances of contrast-induced nephropathy or major complications among the 90 patients enrolled. Lastly, it is important to mention the skepticism of some interventional cardiologists regarding the use of OCT during P-PCI, as it is perceived as somewhat cumbersome, requiring a significant increase in procedural time during emergencies, especially during night shifts or in Cath Labs where there is no second operator adequately trained in the use of the console and the preparation of the OCT catheter.

## 5. The Role of OCT in AMI Tailored Therapy

### 5.1. DAPT

In the management of ACS, DAPT comprising low-dose aspirin and a potent P2Y12 inhibitor such as prasugrel or ticagrelor is recognized as the cornerstone of antithrombotic treatment [145]. As highlighted in the recent literature, the variability inherent in clinical settings, patient characteristics, and individual responses to pharmacotherapy underscores the importance of personalized medicine in determining a patient-specific antithrombotic regimen [146]. The standard DAPT regimen is generally prescribed for a duration of 12 months; however, this timeline can be tailored to meet the specific needs of the patient. For individuals classified as high bleeding risk (HBR), a short duration of dual antiplatelet DAPT may be considered, potentially lasting as little as one month after the ACS [12]. Conversely, patients who are not at high bleeding risk but present with elevated ischemic risk may benefit from an extension of DAPT beyond the typical 12-month period. Recent guidelines from the ESC [12] further emphasize the determinants of high thrombotic risk in patients with ACS. These technical aspects are better evaluated by OCT and include the implantation of at least three stents, treatment of three or more lesions, a total stent length exceeding 60 mm, a history of complex revascularization procedures (such as left main stenting, bifurcation stenting with two or more stents, or chronic total occlusion), and any previous incidents of stent thrombosis despite antiplatelet therapy [12].

The selection of antiplatelet agents further exemplifies the necessity of individualized treatment approaches. In instances where patients present with a heightened bleeding risk, the use of clopidogrel is often prioritized, especially when more potent P2Y12 inhibitors are unavailable [147]. 

In this context, OCT plays a pivotal role by providing critical insights into the characteristics of atherosclerotic plaques. This advanced imaging technique allows for the assessment of plaque stability—whether the plaque is fissured or eroded—and helps in the identification of calcific nodules and the correct positioning of stents [14]. Such detailed evaluation is vital for reclassifying patients who may be at an increased thrombotic risk, thereby guiding clinicians in their decisions regarding the most appropriate antithrombotic therapies [9].

When plaque instability is confirmed, even through OCT imaging, or when it is suspected to be the pathogenic mechanism, the recommended management approach involves administering statin therapy alongside a one-year course of DAPT, which is subsequently followed by single antiplatelet therapy (SAPT). This recommendation holds true even in the presence of mild atherosclerosis [148]. Furthermore, decisions regarding stent implantation should remain contingent upon the characteristics of individual lesions [148].

In cases where non-obstructive PE is detected, a conservative antiplatelet therapy consisting of aspirin and ticagrelor may be considered. Studies have demonstrated this regimen’s effectiveness in preventing MACE while mitigating the complications associated with stent implantation [149,150].

Conservative strategies are also advocated for patients with SCAD [12], where PCI should be reserved for those presenting with hemodynamic instability, left main artery involvement or ongoing ischemia, given the substantial complication rates associated with revascularization procedures [140].

The consideration of antiplatelet therapy in patients with SCAD who do not undergo PCI remains contentious. Previous consensus favored a DAPT regimen in these instances [151]; however, recent findings from the DISCO study suggest that patients on SAPT are at an increased risk of adverse CV events compared to those on DAPT, thus complicating the decision-making process [152].

The assessment of moderate-to-severe calcified coronary (MSCC) lesions via OCT may represent an important factor in guiding the duration of DAPT. A study by Lin et al. [153], involving 1730 patients, categorized by DAPT duration—one year or less versus more than one year—revealed that prolonged DAPT significantly reduced the risk of MACE and cerebrovascular events [153]. With a notable reduction in all-cause and CV mortality rate, this research underscores the significance of long-term DAPT, particularly in patients with MSCC, as it diminishes ischemic events while upholding safety standards [153]. In conclusion, the precise characterization of lesions provided by OCT is instrumental in tailoring a personalized DAPT strategy, ensuring an appropriate balance between thrombotic risk and therapeutic efficacy, particularly within the context of acute coronary syndromes. The integration of advanced imaging techniques and the application of personalized medicine hold the key to enhancing patient outcomes in this complex clinical arena.

### 5.2. Lipid-Lowering Therapies

Lowering low-density lipoprotein cholesterol (LDL-C) following an ACS event significantly reduces CV event rates. The 2023 ESC guidelines for ACS management recommend lowering LDL-C to below 1.4 mmol/L and achieving at least a 50% reduction from the baseline for secondary prevention. For patients who experience a second CV event within two years, the goal is to reduce LDL-C further, to below 1.0 mmol/L. High-intensity statin therapy should be initiated as early as possible after an ACS event, aiming for the highest tolerated dose to achieve LDL-C targets (Class I). If patients were on low- or moderate-intensity statins prior to the event, the intensity should be increased (Class I). If LDL-C targets are unlikely to be met with statins alone, ezetimibe should be added during hospitalization (Class IIb). If patients were on the highest tolerated lipid-lowering therapy (LLT) (statin dose or statin and ezetimibe) prior to the ACS event but with LDL-C levels above the target, adding ezetimibe and a PCSK9 inhibitor is recommended (Class I) [12]. LDL-C should be rechecked 4–6 weeks after treatment adjustments; if LDL-C goals are not achieved with the highest tolerated statin dose, it is recommended to add ezetimibe, and if targets are still unmet after 4–6 weeks, to initiate a PCSK9 inhibitor (Class I) [12]. Among traditional CV risk factors, lipid metabolism plays a central role, and LLTs are an established intervention for both primary and secondary CV prevention. Several studies have shown that these therapies, by reducing LDL-C levels, significantly lower the risk of CV events. It is now well-established that the risk of recurrent ischemic events is closely linked to the morphology of atherosclerotic plaques, which influences the natural history of atherosclerosis. A large plaque burden, a thin fibrous cap with a large lipid arc, small MLA, and macrophages are vulnerable plaque characteristics associated with an increased risk of recurrent events in patients with ischemic heart disease [48,89,93,154]. Some studies have highlighted that LLTs, by reducing LDL-C levels, have beneficial effects on plaque phenotype by reducing the plaque burden and lipid-necrotic core volume and increasing the fibrous cap thickness. Several studies have demonstrated the clinical effectiveness of LLT in patients with both stable CAD [155,156,157] and ACS [156,158,159,160,161,162,163,164], where vulnerable plaques were assessed through serial intracoronary imaging at both baseline and follow-up. These studies have shown that LLT modifies plaque phenotype by increasing fibrous cap thickness and reducing the size of the lipid-necrotic core, thereby making the plaque more stable and preventing the recurrence of CV events. These findings underscore the critical importance of aggressive lipid management in stabilizing atherosclerotic plaques and reducing the risk of future CV events. In this scenario, OCT emerges as a potentially valuable tool for guiding LLT by providing detailed insights into plaque phenotype and vulnerability. However, it is important to note that, despite its potential, there are currently no formal recommendations for adjusting LLT based on plaque phenotype as assessed by OCT. This highlights the need for further research to explore the role of plaque morphology and the mechanism of ACS in tailoring treatment strategies.

### 5.3. Anti-Inflammatory Therapy

A growing body of experimental and clinical evidence supports the central role of inflammation in both the development of atherosclerosis and the pathophysiology of ischemic events [165,166,167]. Markers of inflammation, such as C-reactive protein (CRP), have been shown to be closely associated with CV disease risk, CAD severity, and the recurrence of CV events, independent of traditional risk factors [165,168]. Inflammation is pivotal in the natural history of atherosclerosis, significantly influencing plaque vulnerability leading to the two primary mechanisms of atherothrombosis: PR and PE [165]. It is now well established that various inflammatory cells play crucial roles in the formation, expansion, and destabilization of atherosclerotic plaques. Among these, macrophages, neutrophils, and lymphocytes are central players [165]. Evidence about the role of inflammatory cells in the context of ACS culprit lesions with intact fibrous caps (IFCs) (e.g., PE) has been provided by the OPTICO-ACS study. It was a prospective multicenter study involving 170 patients diagnosed with ACS, focusing on those with intact fibrous cap (IFC) lesions [169]. Patients underwent PCI, with integration of OCT imaging and flow cytometry for comprehensive analysis. The study aimed to explore the microenvironment around the culprit lesions and their associated immune mechanisms. The key results indicated that 24.6% of the ACS cases were attributed to IFC lesions, which exhibited distinct characteristics such as lower lipid content and a thicker fibrous cap when compared to ruptured fibrous cap (RFC) lesions [169]. Notably, the microenvironment of IFC lesions showed significantly elevated concentrations of CD4+ and CD8+ T-lymphocytes, alongside effector molecules like granzyme A, suggesting an immune-mediated process in PE. In vitro experiments demonstrated that CD8+ T-lymphocytes could induce endothelial cell death, particularly under disturbed laminar flow conditions typical at coronary bifurcations [169]. This evidence underscores the critical role of adaptive immunity in the pathogenesis of ACS with IFC culprit lesions, pointing towards new therapeutic targets for the better management of these patients.

LLT, a cornerstone in the pharmacotherapy of CAD, exhibits anti-inflammatory effects. Beyond their lipid-lowering effects, statins have notable anti-inflammatory properties. They reduce the release of CRP, chemokines, cytokines, and adhesion molecules, and modulate T-cell activity. Several studies have highlighted that the anti-inflammatory effect of statins, as measured by CRP levels, prevent the recurrence of CV events irrespective of LDL reduction [170] and that CRP levels decrease independently of LDL levels [171]. Recent clinical trials have investigated the potential role of anti-inflammatory therapy in reducing CV events in patients already receiving standard-of-care treatments, including statins. The CANTOS trial [172] has shown that anti-inflammatory therapy with canakinumab, an antagonist of interleukin-1β, in patients with a history of AMI and high-sensitivity CPR levels of ≥2 mg/L, was associated with a modest reduction of the rate of MACEs compared to placebo, independent of lipid-level lowering. Notably, the study highlighted the importance of inflammation as a therapeutic target, independent of lipid-lowering strategies. The COLCOT trial [173] and LoDoCo2 trial [174] have demonstrated the efficacy of colchicine in reducing CV events in ACS and stable CAD patients already receiving full standard-of-care treatment, including high-dose statins. Based on the COLCOT and LoDoCo2 trials, the COLOCT study [25] investigated the effects of colchicine on atherosclerotic plaques using OCT. A total of 128 patients with ACS and lipid-rich plaques, assessed via OCT, were randomized to receive either colchicine or a placebo for 12 months. Compared to the placebo, colchicine therapy significantly increased the minimal fibrous cap thickness and reduced the average lipid arc, mean angular extension of macrophages, levels of high-sensitivity CRP, interleukin-6, and myeloperoxidase [25]. The evidence from this study, reinforcing the inflammation hypothesis of atherosclerosis, suggests that colchicine has a favorable effect on plaque stabilization [25], similar to what has been observed with LLT. This provides the basis for the future consideration of colchicine or other anti-inflammatory drugs in managing patients with ACS, alongside traditional lipid-lowering treatments. Additionally, OCT could, in future, serve as a useful guide in medical therapy by identifying vulnerable plaques that might benefit from anti-inflammatory treatments.

## 6. Conclusions

This narrative review highlights the crucial role of OCT in the interventional management of myocardial infarction. Thanks to its extraordinary spatial resolution, OCT is an indispensable diagnostic tool, as it allows for the identification of the underlying mechanism of ACS, distinguishing between PR, PE, and CNs, and it surpasses other modalities in quantifying and characterizing thrombi. Moreover, it is essential in the evaluation of NCLs in cases of MVD, providing a comprehensive view of the patient’s CAD. OCT enables the detailed morphological assessment of atherosclerotic lesions and can detect a range of features related to atherosclerotic disease vulnerability, contributing to the definition of atherosclerotic disease vulnerability. This insight should be integrated with clinical, instrumental, and procedural data to identify “vulnerable” patients who may benefit from personalized secondary prevention therapies. Additionally, OCT is a fundamental guide for P-PCI, allowing for detailed procedural planning and, most importantly, enabling the verification and optimization of stent deployment, thereby improving the long-term effectiveness of interventional treatment. Finally, thanks to the valuable insights it provides, OCT has the potential in the future to guide clinicians toward increasingly personalized post-infarction therapy. This could lead to the optimization of antithrombotic, lipid-lowering, and, when necessary, anti-inflammatory therapies, further improving patient clinical management.

## Data Availability

All data generated or analyzed during this study are included in this published article. Further inquiries should be directed to the corresponding author.

## Abbreviations

ACS	Acute Coronary Syndrome
AI	Artificial Intelligence
AKI	Acute Kidney Injury
AMI	Acute Myocardial Infarction
AMD	Age-Related Macular Degeneration
ARC	Academic Research Consortium
ASM	Acute Stent Malapposition
BVS	Bioresorbable Vascular Scaffolds
CC	Cholesterol Crystal
CIN	Contrast-Induced Nephropathy
CKD	Chronic Kidney Disease
CN	Calcified Nodule
CRP	C-Reactive Protein
CSCR	Central Serous Chorioretinopathy
CV	Cardiovascular
DAPT	Dual Antiplatelet Therapy
DES	Drug-Eluting Stents
DM	Diabetes Mellitus
EAPCI	European Association of Percutaneous Cardiovascular Intervention
ESC	European Society of Cardiology
FD	Fourier Domain
FFR	Fractional Flow Reserve
GDMT	Guideline-Directed Medical Therapy
HBR	High Bleeding Risk
ISR	In-Stent Restenosis
IVUS	Intravascular Ultrasound
IVUS-VH	IVUS Virtual Histology
LAD	Left Anterior Descending Artery
LASM	Late Acquired Stent Malapposition
LDL-C	Low-Density Lipoprotein Cholesterol
LLT	Lipid-Lowering Therapies
LPSM	Late Persistent Stent Malapposition
LSM	Late Stent Malapposition
MACE	Major Adverse Cardiovascular Events
MSE	Major Safety Events
MINOCA	Myocardial Infarction with Non-Obstructive Coronary Arteries
MLA	Minimum Lumen Area
MSA	Minimum Stent Area
MSCC	Moderate-to-Severe Calcified Coronary
MVD	Multivessel Disease
NCLs	Non-Culprit Lesions
NIRS	Near-Infrared Spectroscopy
NSTEACS	Non-ST-Segment Elevation ACS
OCT	Optical Coherence Tomography
P-PCI	Primary PCI
PCI	Percutaneous Coronary Intervention
PE	Plaque Erosion
PR	Plaque Rupture
RCTs	Randomized Controlled Trials
SAPT	Single Antiplatelet Therapy
SCAD	Spontaneous Coronary Artery Dissection
SD	Spectral Domain
SM	Stent Malapposition
SS-OCT	Swept Source OCT
ST	Stent Thrombosis
STEMI	ST-Segment Elevation MI
TCFA	Thin-Cap Fibroatheroma
